# Gender Differences in Ghrelin Association with Cardiometabolic Risk Factors in Arab Population

**DOI:** 10.1155/2014/730472

**Published:** 2014-09-09

**Authors:** Mohamed Abu-Farha, Mohammed Dehbi, Fiona Noronha, Ali Tiss, Monira Alarouj, Kazem Behbehani, Abdullah Bennakhi, Naser Elkum

**Affiliations:** ^1^Biochemistry and Molecular Biology Unit, Dasman Diabetes Institute, P.O. Box 1180, Kuwait City, Kuwait; ^2^Diabetes Research Center, Qatar Biomedical Research Institute, P.O. Box 5825, Doha, Qatar; ^3^Biostatistics and Epidemiology Department, Dasman Diabetes Institute, P.O. Box 1180, Kuwait City, Kuwait; ^4^Dasman Diabetes Institute, P.O. Box 1180, Kuwait City, Kuwait; ^5^Clinical Epidemiology, Sidra Medical and Research Center, P.O. Box 26999, Doha, Qatar

## Abstract

Ghrelin is a stomach produced hormone that has been shown to have protective role against development of CVD which is a leading cause of death in the Arab world. The objective of this study is to examine the gender difference in association between traditional CVD risk factors and plasma ghrelin among Arabs. 359 Arab residents in Kuwait participated in a cross-sectional survey (≥20 years old): 191 were females and 168 were males. Plasma level of ghrelin was assessed using Luminex-based assay. Ghrelin levels were significantly higher in females (935 ± 78 pg/mL) than males (763 ± 65 pg/mL) (*P* = 0.0007). Females showed inverse association with WC (*r* = −0.23, *P* = 0.001) and HbA1C (*r* = −0.19, *P* = 0.0102) as well as SBP (*r* = −0.15, *P* = 0.0383) and DBP (*r* = −0.16, *P* = 0.0230), respectively. Higher levels of ghrelin were shown to associate with increased insulin resistance, as measured by HOMAIR, in male Arab subjects (*P*-trend = 0.0202) but not in females. In this study we show that higher ghrelin level was negatively associated with measures of obesity, HbA1C, and blood pressure in females and positively associated with increased insulin resistance in Arab males.

## 1. Introduction

Ghrelin is a 28-amino-acid peptide hormone that is mainly secreted by the stomach mucosa [1]. It has been shown to play a major role in the activation of the growth hormone secretagogues receptor 1a (GHS-R1a) causing the release of growth hormone (GH) [2]. Ghrelin is very efficient at the release of GH causing an increase in circulating plasma GH level upon its injection in humans and rats peaking after 15–20 min and lasting over 60 min [3, 4]. In addition to its ability to release GH, ghrelin is involved in appetite stimulation, regulation of energy homeostasis, immune response, and cardiovascular activities [3, 5, 6]. Its roles in the cardiovascular system include cardioprotective activity against ischemia, improvement of vasodilatation, and regulation of blood pressure (BP) [4]. Intravenous administration of ghrelin has been proposed as an adjuvant therapy as it increases left ventricular mass and ejection fraction in patients with chronic heart failure [7]. On the other hand, its administration to normal subjects increases cardiac contractility and output in addition to decreasing mean arterial pressure. It is believed that these beneficial effects of ghrelin are due to its ability to protect endothelial cells by inhibiting proinflammatory processes and angiotensin II induced migration of these cells as well as its antagonist action on vasoconstrictor endothelin-1 and increased acetylcholine induced vasorelaxation or increase in endothelial nitric oxide synthase [5]. As a result higher levels of ghrelin are believed to be associated with reduced cardiovascular disease (CVD) risks [4, 5].

Driven by increase in food uptake and sedentary life style, increasing prevalence of CVDs is expected to increase the burden on health care systems worldwide [8]. Most CVDs are associated with risk factors such as hypertension, obesity, hyperlipidaemia, and type 2 diabetes (T2D) [9]. Women are known to live longer and develop CVDs at a later age [10] and have a lower prevalence of metabolic syndrome than men [10]. Interestingly, females are known to have higher level of ghrelin than males [11]. Even though CVDs are a leading cause of death in the Arab world, gender differences in the association between metabolic markers, such as ghrelin and CVD risk factors, are not thoroughly studied in the Arab population [12]. To investigate gender difference in ghrelin in this population, we focused on the association between ghrelin and traditional metabolic risk factors such as fasting blood glucose (FBG), glycated haemoglobin (HbA1c), fasting insulin, and the lipid profile that included triglycerides (TG), total cholesterol (TC), low-density lipoprotein (LDL), and high-density lipoprotein (HDL), in addition to BP and ghrelin in adult Arabs. To the best of our knowledge, this is the first report about the link of the ghrelin and CVD risk factors in Arabs and to investigate the gender difference in its association with ghrelin plasma level.

## 2. Materials and Methods

### 2.1. Study Participants and Ethical Considerations

In this study, a cross-sectional population-based survey was conducted on 359 adult Arab expatriates living in Kuwait. A full description of the study design and data collection was outlined previously [13]. Briefly, a random representative subgroup of our cohort including 191 females and 168 males was selected. The subjects were randomly selected from the computerized register of the Public Authority of Civil Information and were originating from different Arab countries including Egypt, Syria, Lebanon, Jordan, Palestine, and Arab gulf countries. All subjects were ≥20 years old. The study conformed to the principles outlined in the Declaration of Helsinki and was approved by the institutional Ethical Review Committee at Dasman Diabetes Institute. An informed written consent was obtained from all the participants before their enrolment in the study. Subjects with CVD were excluded from the study as outlined previously [14]. History of medication was recorded as shown previously [13]. This study was carried out between June 2011 and August 2012.

### 2.2. Anthropometric and Physical Measurements

Physical and anthropometric measurements included body weight, height, hip, and waist circumferences (WC) as well as systolic blood pressure (SBP) and diastolic blood pressure (DBP). BP was measured with Omron HEM-907XL Digital sphygmomanometer. An average of 3 BP readings, with 5–10 minutes rest between each, was obtained. Height and weight were measured using calibrated portable electronic weighing scales and portable inflexible height measuring bars. Participants were wearing light indoor clothing and bear-footed during the measurement process. Hip and WC were measured using constant tension tape at the end of a normal expiration, with arms relaxed at the sides, the highest point of the iliac crest and the midaxillary line. Body mass index (BMI) was calculated using the standard formula: body weight (in kilograms) divided by height (in meters squared).

### 2.3. Clinical Laboratory Measurements

Blood samples were obtained after an overnight fasting for at least 10 hours and analyzed for FBG, HbA1c, fasting insulin, and lipid profiles that included TG, TC, LDL, and HDL. Glucose and lipid profiles were measured on the Siemens Dimension RXL chemistry analyzer (Diamond Diagnostics, Holliston, MA). HbA1c was determined using the Variant device (BioRad, Hercules, and CA). All laboratory tests were performed by certified technicians at the clinical laboratories of DDI, using the Ministry of Health approved methods and quality standards. Insulin resistance was calculated using the International Homeostasis Model Assessment Insulin Resistance (HOMA-IR) formula:

(1) $$\begin{array}{c} \text{HOMA-IR}=\frac{\text{FBG}\left(\text{mmol}/\text{L}\right)\times \text{fasting}\;\;\text{Insulin}\left(\text{mU}/\text{L}\right)\;}{22.5}. \end{array}$$

### 2.4. Ghrelin Plasma Measurements

To measure ghrelin, blood was drawn into vacutainer EDTA aprotinin tubes. Plasma was obtained after centrifugation, aliquoted, and then stored at −80°C. Plasma samples stored at −80 freezers were thawed on ice and used to measure level of acylated ghrelin. Plasma levels of acylated ghrelin were assessed using the multiplexing immunobead array platform according to the manufacturer instructions (BioRad, Hercules, CA). Experimental data was processed with Bio-Plex manager software version 6 (Bio-Rad, Hercules, CA) using five-parametric curve fitting. Intra- and interplate coefficients of variation (CV) ranged from 6% to 25%, respectively. Samples were measured using reagents from the same batch to avoid batch-to-batch variations.

### 2.5. Statistical Analysis

Normality tests were run to assess data distribution. Since ghrelin concentration was skewed, the values were log-transformed before data analysis. Categorical variables are reported as numbers and percentages, continuous variables are reported as means with standard deviations (SD), and ghrelin is reported as median with range. Differences between groups were analyzed with Chi-square tests for categorical variables, unpaired *t*-tests for normally distributed continuous variables, and Mann-Whitney test for ghrelin concentration levels. Spearman's correlation analysis was performed between ghrelin and classical CVD risk factors. Subjects in the overall population were classified into tertiles based on their circulating HbA1C levels (*T*_1_ < 5.3, 5.3 ≤ *T*_2_ ≤ 6.0, and *T*_3_ > 6.0), waist to hip ratios (*T*_1_ < 0.88, 0.88 ≤ *T*_2_ ≤ 0.94, and *T*_3_ > 0.94), and HOMA-IR (*T*_1_ < 1.53, 1.53 ≤ *T*_2_ ≤ 3.01, and *T*_3_ > 3.01). Furthermore, subjects were classified according to their BMI as lean, overweight, and obese (BMI between 18.5 and 24.9 was considered lean, 25 and 29.9 was considered overweight, ≥30 was considered obese). In this analysis ghrelin was log-transformed and was expressed as the geometrical mean. Research Electronic Data Capture (REDCap) was used for data collection and data management. All statistical assessments were two-sided and considered to be significant when *P* value < 0.05. All the analyses were performed using SAS (version 9.2; SAS Institute, Cary, NC).

## 3. Results

### 3.1. Population Characteristics and Anthropometric Measurements

The characteristics of population analyzed in this study are shown in Table 1. The total number of subjects interviewed was 359 with the age ≥20 years; of which 53.2% were females (191 subjects) and 46.8% were males (168 subjects). Both females and males had similar mean of age (44.2 year ± 11.8 and 44.9 year ± 11.1, resp.) as shown in Table 2. Females had significantly higher BMI than males (*P* < 0.0001); whereas, WC was higher in males than in females (*P* = 0.0186). SBP, DBP, FBG, TG, and HbA1C were shown to be lower in females when compared to males (*P* < 0.05). However, males had a significantly lower HOMA-IR, TC, HDL, LDL, and ghrelin (*P* < 0.05) when compared to females.

### 3.2. Spearman's Correlation between Ghrelin and Cardiometabolic Risk Factors

Spearman's correlation showed significant inverse association between ghrelin and waist to hip ratio (*r* = −0.23, *P* = 0.0011) in females. Significant inverse association was also shown for HbA1C (*r* = −0.19, *P* = 0.0102), SBP (*r* = −0.15, *P* = 0.0383), and DBP (*r* = −0.16, *P* = 0.0230). On the other hand, Arab males showed no significant Spearman's correlation between plasma level of ghrelin and any of the measured cardiometabolic risk factors. BMI showed weak but not statistically significant inverse association with ghrelin in both males and females (*r* = −0.14, *P* = 0.0663) and (*r* = −0.14, *P* = 0.0534), respectively. Using the whole population, ghrelin demonstrated significant inverse correlations with waist to hip ratio (*r* = −0.21, *P* ≤ 0.0001) and HbA1C (*r* = −0.19, *P* = 0.0002) as well as SBP (*r* = −0.11, *P* = 0.0318) and DBP (*r* = −0.12, *P* = 0.0203).

### 3.3. Ghrelin Plasma Levels and the Effect of Age Distribution

**Figure 1:**
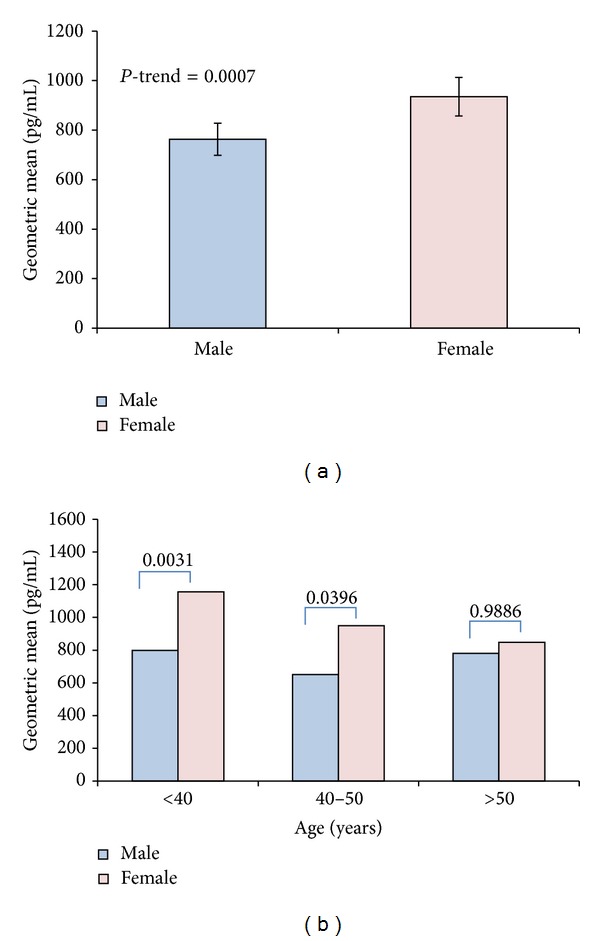
Ghrelin level in male and female Arab subjects. (a) Difference in plasma level of ghrelin in female and male Arab subjects reported as least square values adjusted for age and BMI. (b) Age distribution of plasma level ghrelin adjusted for BMI between male and female subjects. Generally, younger females less than 40 years old had the highest ghrelin level, which was decreasing with age.

Figure 1 presents the age and BMI adjusted least square means of the circulating levels of ghrelin in males and females. Overall, females have higher circulating levels of ghrelin (*P* = 0.0007). Least square geometric mean of ghrelin in males was 763 (65) pg/mL while females had 935 (78) pg/mL as shown in Figure 1(a). To investigate the effect of age on the level of ghrelin, our population was divided into three groups (under 40, 40–50, and over 50 years old) as shown in Figure 1(b). In general, least square means of the circulating levels of ghrelin in females was higher than males except for above 50 years age group, where there was no difference between the two genders (*P* = 0.9886). Females had a significantly higher level of ghrelin, when compared to males, in the under 40 years age group (*P* = 0.0031) as shown in Figure 1(b). The group between 40 and 50 years of age showed a similar trend but lower levels of ghrelin as shown in Figure 1(b).

### 3.4. Ghrelin Plasma Levels and Their Distribution according to HbA1C Tertiles

**Figure 2:**
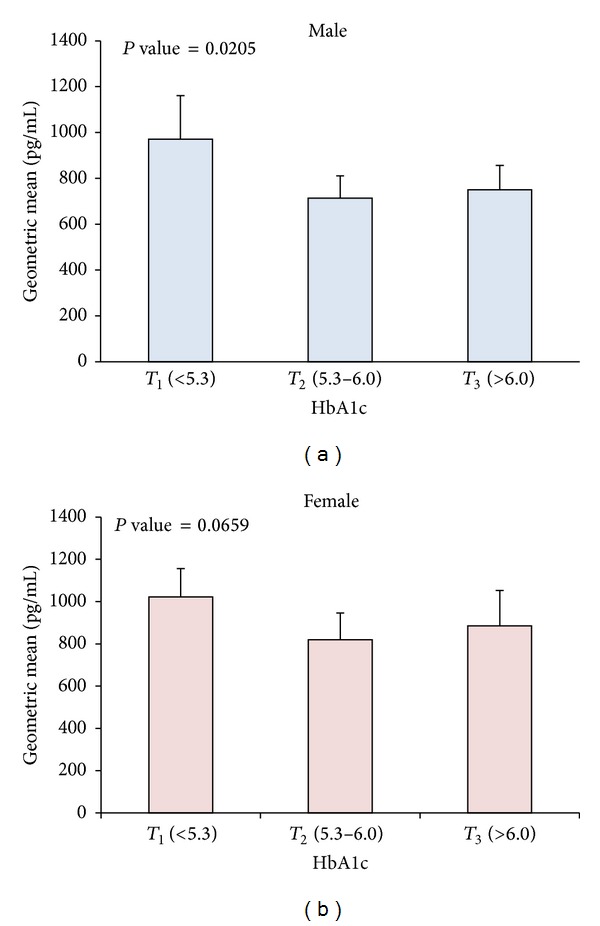
Age and BMI adjusted geometric means of ghrelin level and their effect on HbA1C. (a) Age and BMI adjusted ghrelin levels according to tertiles of HbA1C (*T*_1_ < 5.3, 5.3 ≤ *T*_2_ ≤ 6.0, and *T*_3_ > 6.0) showing association between decreasing level of HbA1C and decreasing ghrelin level in males (*P*-trend = 0.0205). (b) Age and BMI adjusted ghrelin levels according to tertiles of HbA1C showing weak but not significant decrease in HbA1C as ghrelin level decreases in females (*P*-trend = 0.0659).

After dividing the volunteers based on HbA1C tertiles (*T*_1_ < 5.3, 5.3 ≤ *T*_2_ ≤ 6.0, and *T*_3_ > 6.0) a significant drop in age and BMI adjusted ghrelin geometric mean between *T*_1_ and *T*_3_ males (*P*-trend = 0.0205) was seen (Figure 2(a)). However, No significant difference in ghrelin level was observed between *T*_2_ and *T*_3_. On the other hand, a similar trend was observed in females which did not reach statistical significance (*P*-trend = 0.0659) as shown in Figure 2(b).

### 3.5. Ghrelin Plasma Levels and Their Distribution according to BMI

**Figure 3:**
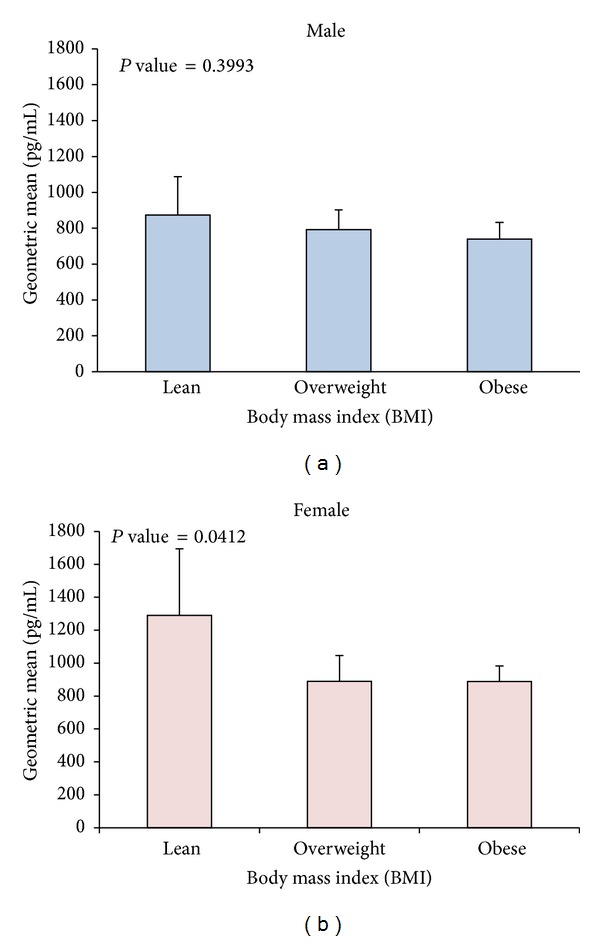
Age adjusted geometric means of ghrelin level and their effect on BMI. (a) Age adjusted ghrelin levels according to different classes of BMI (BMI between 18.5 and 24.9 was considered lean, 25 and 29.9 was considered overweight, ≥30 was considered obese) showing no significant change in ghrelin level across the different BMI classes in Arab males. (b) Females on the other hand showed significant decrease in ghrelin level in obese subjects compared to lean and overweight (*P*-trend = 0.0412).

To look at the ghrelin distribution across different BMI groups (BMI between 18.5 and 24.9 was considered lean, 25 and 29.9 was considered overweight, ≥30 was considered obese), the population was divided into three groups according to BMI (lean, overweight, and obese). Males did not show any difference in ghrelin levels across the different BMI classes as shown in Figure 3(a). Age adjusted geometric means of ghrelin level showed significant difference between lean, overweight, and obese in females (*P*-trend = 0.0412) as shown in Figure 3(b).

### 3.6. Ghrelin Plasma Levels and Their Distribution according to Waist to Hip Ratio Tertiles

**Figure 4:**
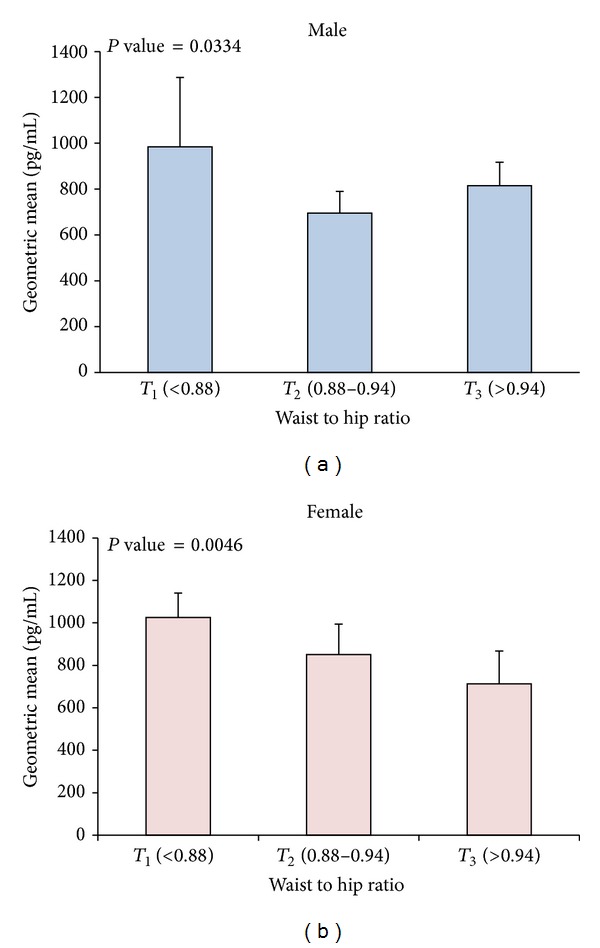
Age and BMI adjusted geometric means of ghrelin level and their effect on waist to hip ratio. (a) Age and BMI adjusted ghrelin levels according to tertiles of waist to hip ratios (*T*_1_ < 0.88, 0.88 ≤ *T*_2_ ≤ 0.94, and *T*_3_ > 0.94) showing association between increasing waist to hip ratio and decreasing ghrelin level in males (*P*-trend = 0.0334). (b) Age and BMI adjusted ghrelin levels according to tertiles of waist to hip ratios showing significant reduction in ghrelin level in subjects with higher waist to hip ratios in females (*P*-trend = 0.0046).

Age and BMI adjusted ghrelin levels according to tertiles of waist to hip ratios (*T*_1_ < 0.88, 0.88 ≤ *T*_2_ ≤ 0.94, and *T*_3_ > 0.94) showed strong significant reduction between *T*_1_ and *T*_3_ in Arab males (*P*-trend = 0.0334) as shown in Figure 4(a). Even though *T*_3_ showed higher ghrelin level than *T*_2_, values were not statistically significant. Females showed a similar trend of reduction in ghrelin level in subjects with higher tertiles of waist to hip ratios (*P*-trend = 0.0046) as shown in Figure 4(b).

### 3.7. Ghrelin Plasma Levels and Their Distribution according to HOMA-IR Tertiles

**Figure 5:**
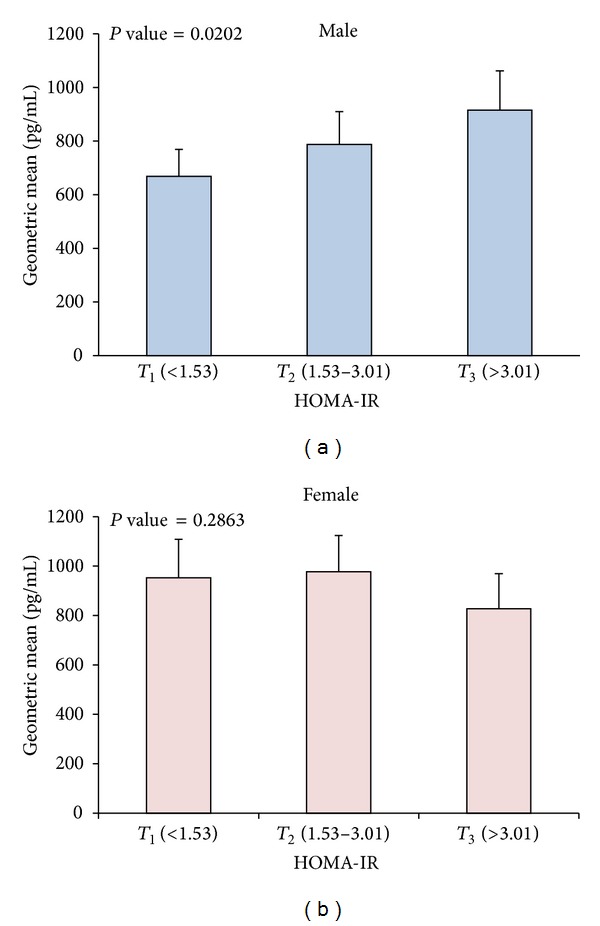
Age and BMI adjusted geometric means of ghrelin level and their effect on HOMA-IR. (a) Age and BMI adjusted ghrelin levels according to tertiles of HOMA-IR (*T*_1_ < 1.53, 1.53 ≤ *T*_2_ ≤ 3.01, and *T*_3_ > 3.01) showing association between increasing insulin resistance and increasing ghrelin level in males (*P*-trend = 0.0202). (b) Age and BMI adjusted ghrelin levels according to tertiles of waist to hip ratios showing no significant change in ghrelin level in subjects with increasing level of insulin resistance as measured by HOMA-IR in females.

Dividing subjects according to tertiles of insulin resistance as measured by HOMA-IR (*T*_1_ < 1.53, 1.53 ≤ *T*_2_ ≤ 3.01, and *T*_3_ > 3.01) showed strong correlation between subjects with higher HOMA-IR levels and increased ghrelin level in Arab males (*P*-trend = 0.0202) as shown in Figure 5(a). Females on the other hand showed no significant change in ghrelin level in higher tertiles of HOMA-IR in this population Figure 5(b).

## 4. Discussion

This study examined the distribution and the association between various established cardiometabolic risk factors and circulating level of ghrelin in an Arab population. It was shown that plasma level of circulating ghrelin was significantly higher in Arab females as compared to males. Also, ghrelin level was shown to be age dependent, showing a significant decrease particularly in female subjects older than 50 years old. In Spearman's correlation, ghrelin levels were negatively associated with waist to hip ratio, HbA1c, SBP, and DBP in Arab females. Adjusting for age and BMI showed that males in highest tertiles of HbA1C and waist to hip ratio had lower level of ghrelin in males and females. Furthermore Arab male subjects showed significant correlation between increased insulin resistance as measured by HOMA-IR and the increased levels of ghrelin.

### 4.1. Correlation between Ghrelin Plasma Levels and Obesity

Circulating levels of ghrelin showed negative correlation with waist to hip ratio and BMI in Arab females. Our data agrees with previously reported data regarding ghrelin level decrease in obese subjects compared to lean controls [15]. It is suggested that this negative correlation is caused by the body's reaction to limit further food intake [16]. As a result, ghrelin level negatively correlates with factors that are increased in obese subjects such as percentage of body fat, insulin, and leptin levels [16]. However, blockage of ghrelin in diet-induced obese mice resulted in reduction of food intake and body weight suggesting that its blockage can protect against obesity [17]. On the other hand, weight loss causes elevation of ghrelin level making further weight loss more difficult [18]. This increase in ghrelin level is an initiator of food intake to increase blood glucose as a result of low blood nutrients levels. This is particularly important in cases of severe caloric restriction to prevent hypoglycemia [19]. Ghrelin plays an important function in appetite regulation and energy homeostasis [4, 20]. Gender difference in ghrelin association with BMI and WC could be due to its differential association with fat distribution in the body. Total ghrelin has been shown to inversely associate with fat cell size [21]. In women, ghrelin negatively associates with total fat and fat mass/lean mass ratio unlike men where it associates with abdominal fat mass and fat distribution index [21].

### 4.2. Gender Difference in Association between Ghrelin Levels and Insulin Resistance as Measured by HOMA-IR in Arab Subjects

Insulin resistance is a primary risk factor in CVDs and other metabolic diseases [4]. Its mechanism is complex and involves many pathways that are not fully understood [4]. In this study we showed that, independently of age and BMI, high ghrelin plasma level was associated with insulin resistance in Arab males as estimated by HOMA-IR but not females as shown in Figure 5. Age and BMI have been suggested as two important confounders in the ghrelin regulation [11, 21, 22]. Our population had a similar mean age unlike BMI which was significantly higher in women. Their effect on the association with HOMA-IR is not very well understood and the association between insulin resistance and ghrelin has been disputed with some studies showing positive association while others are showing negative association with insulin resistance [16, 23–26]. Ghrelin is present in two forms in circulation, acylated ghrelin (AG), and desacylated ghrelin (DAG) [27]. AG was believed to be the only active form as it displayed strong GH releasing through its binding to GHS-R as well as its stimulation of food intake. For instance, Pöykkö et al. showed that type 2 diabetes patients had lower levels of ghrelin compared to nondiabetic patients independently of age, gender, and BMI [28]. In a different study, strong inverse correlation was observed between ghrelin concentration and insulin levels as well as insulin resistance in middle-aged obese subjects [24] or females with polycystic ovary syndrome [29]. Generally, AG level has been associated with insulin resistance, obesity, and type 2 diabetes [30]. On the other hand, the DAG form has been associated with insulin sensitivity and reduced fat mass in addition to its ability to modulate energy balance in normal conditions [27]. The difference in the function of the two forms has prompted the need for measuring AG/DAG ratios where this ratio is higher in insulin resistance subjects [30]. Our data sheds light on the gender and ethnic difference in the role ghrelin might play in modulating insulin resistance.

### 4.3. Association between Ghrelin and Blood Pressure

Our data shows that ghrelin negatively correlated with SBP and DBP in Arab Females. Several studies looked at the effect of intravenous injection of human ghrelin on BP and heart rate [25, 31–34]. For example, Nagaya et al. showed that intravenous injection of ghrelin caused a long-lasting GH release as well as favorable hemodynamic effects causing a decrease in resistance of peripheral artery that ultimately caused a drop in BP without increasing heart rate [35]. It was also reported that normal pregnant females showed negative correlation between circulating ghrelin level and systemic BP [36]. A recent report also showed negative correlation between ghrelin plasma level and pulmonary arterial pressure in atrial septal defect patients [37]. Many mechanisms of these vasodilatory effects of ghrelin have been proposed based on its antagonist action on vasoconstrictor endothelin-1, increased acetylcholine induced vasorelaxation, or increase in endothelial nitric oxide synthase [5]. This lack of association with BP in males could also be linked to the association between higher ghrelin and increased insulin resistance, more data is required to further show this point. Taken together, our data agrees with the known function of ghrelin and exhibits a similar pattern in Arab population females. The lack of association between SBP and DBP in males highlights the ethnic difference and is worth being further investigated in future studies. Indeed, the cross-sectional design used here makes it impossible to determine any causal relationship between ghrelin and CVDs. A further study is currently underway to prospectively follow up this cohort to determine directionality of the observed associations. However, the strength of our study includes the population-based sample and comprehensive assessment of CVD risk factors in relation to ghrelin. Our study also highlighted the gender difference in our population showing the different role ghrelin can play in both genders.

## 5. Conclusions

In this study we have examined the association between a number of CVD risk factors with the stomach produced hormone ghrelin in male and female Arabs. We showed that, in females, ghrelin might possess a cardioprotective role as it negatively associates with obesity measures, HbA1C, SBP, and DBP. Ghrelin also showed gender difference in its association with insulin resistance; it had positive association in males but not females. In summary, our data exhibit gender difference in ghrelin association with cardiometabolic risk factors and its inverse correlation with reduction of HbA1C in addition to its association with SBP and DBP reduction in female Arab population.

## Figures and Tables

**Table 1:** Clinical and sociodemographic characteristics of the study population.

Characteristics	All (*n* = 359)	Female (*n* = 191)	Male (*n* = 168)	*P* value
Age (years)	44.4 ± 11.5	44.2 ± 11.8	44.9 ± 11.1	0.5481
BMI (kg/m^2^)	31.6 ± 0.34	33.3 ± 0.48	30.6 ± 0.45	<0.0001
Waist circumference (cm)	101.2 ± 0.75	99.39 ± 1.03	103.1 ± 1.09	0.0186
SBP (mmHg)	129.8 ± 1.01	124.8 ± 1.41	135.1 ± 1.36	<0.0001
DBP (mmHg)	78.3 ± 0.64	76.89 ± 0.90	79.77 ± 0.90	0.0242
FBG (mmol/l)	6.23 ± 0.16	5.93 ± 0.18	6.57 ± 0.27	0.0366
HbA1c (%)	6.04 ± 0.09	5.67 ± 0.10	6.46 ± 0.15	<0.0001
HOMA-IR	3.31 ± 0.51	3.46 ± 0.34	3.13 ± 0.23	0.5621
TC (mmol/l)	5.20 ± 0.06	5.32 ± 0.08	5.08 ± 0.09	0.0316
TG (mmol/l)	1.70 ± 0.06	1.54 ± 0.06	1.88 ± 0.11	0.0050
HDL (mmol/l)	1.13 ± 0.02	1.26 ± 0.03	0.99 ± 0.02	<0.0001
LDL (mmol/l)	3.36 ± 0.05	3.42 ± 0.07	3.30 ± 0.08	0.2122
Ghrelin (pg/ml)	860 (240–5360)	960 (240–4090)	750 (290–5360)	0.0049

BMI: body mass index; SBP: systolic blood pressure; DBP: diastolic blood pressure; FBG: fasting blood glucose; TC: total cholesterol; TG: triglycerides; HDL: high-density lipoprotein; LDL: low-density lipoprotein.

Results are reported as mean ± SE except for nonnormally distributed ghrelin levels that are presented as median (range).

**Table 2:** Spearman correlation of ghrelin marker with cardiometabolic risk factors.

Variables	All		Male		Female	
	*ρ*	*P* value	*ρ*	*P* value	*ρ*	*P* value
Age (years)	−0.07	0.1916	−0.03	0.7234	−0.11	0.1438
BMI (kg/m^2^)	−0.10	0.0609	−0.14	0.0663	−0.14	0.0534
Waist to hip	−0.21	<0.0001	−0.02	0.8160	−0.23	0.0011
TC (mmol/l)	0.07	0.1786	0.09	0.2456	0.01	0.8626
TG (mmol/l)	−0.07	0.2017	−0.07	0.3294	−0.03	0.6688
LDL (mmol/l)	0.09	0.0936	0.11	0.1262	0.04	0.6003
HDL (mmol/l)	0.07	0.1674	0.01	0.9263	0.04	0.5725
FBG (mmol/l)	−0.07	0.1800	−0.01	0.9455	−0.10	0.1886
HBA1C (%)	−0.19	0.0002	−0.12	0.1183	−0.19	0.0102
HOMAIR	−0.02	0.6731	0.07	0.3988	−0.12	0.1121
Systolic (mmHg)	−0.11	0.0318	0.02	0.7526	−0.15	0.0383
Diastolic (mmHg)	−0.12	0.0203	−0.03	0.7151	−0.16	0.0230

BMI: body mass index; SBP: systolic blood pressure; DBP: diastolic blood pressure; FBG: fasting blood glucose; TC: total cholesterol; TG: triglycerides; HDL: high-density lipoprotein; LDL: low-density lipoprotein.
